# COVID-19 Is Transforming Economic Policy in the United States

**DOI:** 10.1007/s10272-021-0979-4

**Published:** 2021-08-05

**Authors:** Claudia Sahm

**Affiliations:** Stay-at-Home Macro (SAHM) Consulting, 231 N Edgewood St, Arlington, VA 22201 USA

## Abstract

Cutbacks in government spending slowed the recovery and led to lasting damage to workers and economic growth.

A global pandemic is bringing the sea change in economic policy that the meltdown of financial markets in 2008 did not. While the shift is incomplete and its success is far from guaranteed, now is the time to reflect on its effects and the challenges ahead.

The economic policy response during the COVID-19 pandemic offers four key lessons: First, the features of the coronavirus crisis are different from and the same as past recessions. Second, we must use lessons learned from economic policies in this crisis to prepare for future recessions. Third, good policies require good administration, which was repeatedly missing during the COVID-19 crisis. Good policy is worthless if it never reaches the people it was intended to serve. Finally, it is time for macroeconomics to upgrade its tools and frameworks, bringing in new ideas and retiring some old ones. That effort will require introspection and collaboration. To remain relevant, change in economics is imperative.

## Differences and similarities shape the policy response now and in the future

COVID-19 hit the United States hard. In mid-March 2020, a $21 trillion economy locked down to keep people safe and hospitals from being overburdened by a new, mysterious killer. Several prominent macroeconomists, such as Mankiw (2020) and Summers (2020), heralded it as the first true supply-side recession, since people could not, in their opinion, spend. They concluded that demand policies in recessions, like stimulus checks, were unhelpful and would spark shortages, wasteful deficit spending and high inflation. They called for targeted relief only for people hardest hit like the unemployed and spending on public health efforts. Those actions were necessary but insufficient. The breadth of the crisis was too broad for such a narrow policy response.

**Claudia Sahm**, Stay-at-Home Macro (SAHM) Consulting, Arlington, VA; and Jain Family Institute, New York, USA.

Yes, spending was restricted more than in past recessions, but people still had to have enough money to keep a roof over their heads and feed their children. Moreover, I argued from the start of the pandemic that we were also living "the mother of all demand shocks" (Sahm, 2020). Millions of workers claimed unemployment insurance each week, and the official unemployment rate hurtled toward 15% in April 2020 (Cox, 2020). The speed and breadth of job losses rivaled only the Great Depression. As shown in Figure 1, nearly half of US families lost income from employment during the crisis. While income loss was most common among low-income families, those with higher incomes were also negatively affected.

Economic hardship was even greater among particular groups. People of color (Spriggs, 2021), low-wage service workers (Gould and Kandra, 2021) and mothers (Heggeness, et al., 2021) were hit especially hard. While it is true that in all recessions some are hurt more than others, the severity of this recession magnified the pain of the hardest hit groups. Moreover, most families, even in the best of times, do not have a sufficient financial buffer to weather a lost paycheck (Bhutta et al., 2020). Without government aid, a collapse in demand was inevitable.

Thankfully, policymakers went big, fast and broad. They recognized the novel and the traditional features of this crisis. In March 2020, Congress enacted the $2.2 trillion Coronavirus Aid, Relief, and Economic Security (CARES) Act. It combined targeted relief, such as an extra $600 per week for the unemployed, and broad-based support in $1,200 stimulus checks per adult. By contrast, during the Great Recession, the American Recovery and Reinvestment Act was under $1 trillion, while the extra weekly payments were $25 and the checks $500 per adult.

The large response in the CARES Act worked. Larrimore et al. (2021) found using Internal Revenue Service records that these two relief programs more than replaced the lost employment income for a typical worker with a large income loss due to the COVID-19 recession and provided jobless benefits. Moreover, the support was greatest for low-wage workers.

The coronavirus crisis had unique features, too. First and foremost, it was caused by a pandemic. COVID-19 drove the crisis from the start and will continue to until its end. In response, Congress allocated billions of dollars in public health efforts to fight the pandemic and develop a vac-

Figure 1

**Many families lost income from work, fewer received jobless benefits**

Percent of families by household income in 2019

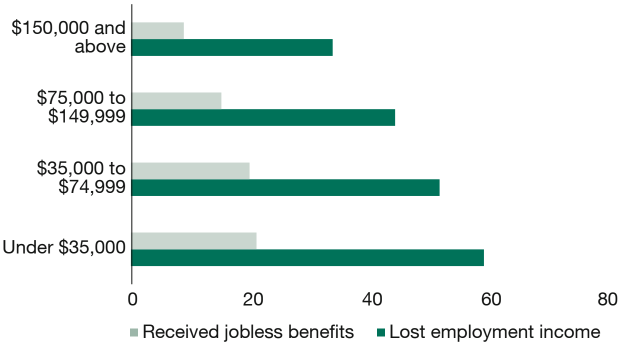

Source: U.S. Census Bureau Householding Pulse Survey for Dec. 9 to Dec. 21, 2020.

Figure 2

**CARES Act relief shielded low-income workers the most from large income losses**

Percent of workers who had large income losses after including relief, by deciles of income in 2019

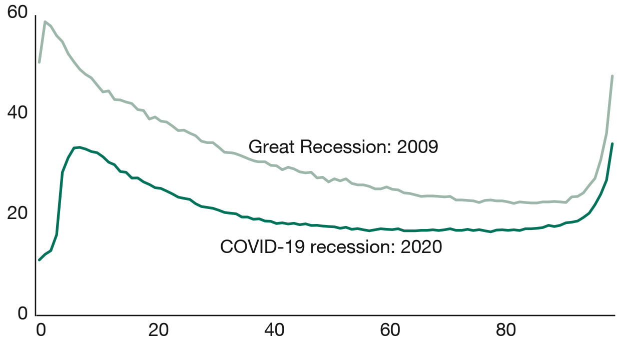

Note: Authors use IRS data from W2 and 1099 forms. Income in 2019 is wages or unemployment insurance. Calculations include workers age 25 or older with income in 2019. Large income loss is a decrease of 10% or more.

Source: Larrimore et al. (2020).

cine. Another difference from the Great Recession is that the fundamentals of the US economy and finances of many families were the best they had been in decades. Policymakers viewed their efforts as a bridge to the other side of the pandemic. So, while income from employment fell broadly, massive relief from the government was able to keep many families afloat.

Some benefits, like enhanced unemployment insurance and stimulus checks, even reached people who were struggling before the coronavirus-induced crisis. Researchers at Columbia University estimate that the American Rescue Plan cut the poverty rate for children from 15% (without relief) to 6% (with the relief); among Black and Hispanic families, the reductions were sizeable, too, from around 20% to 10% (Parolin et al., 2021). Fiscal relief worked, and it worked most for groups who entered the COVID-19 crisis with the most economic challenges and who were hit hardest by the crisis.

## Use lessons from economic policies in this crisis to prepare for future recessions

Go bigger, go faster, go broader – was the lesson from the Great Recession when the policy response fell short. Congress and the Federal Reserve delivered in the spring of 2020. The $2 trillion CARES Act was twice the package that was offered in 2009. Support from Congress waned last summer as COVID-19 cases decreased but then the surge in infection rates in late autumn and into the winter led to another $1 trillion in fiscal support at the end of 2020.

The big test remained. How committed are policymakers to pushing a rapid recovery? In the years following the Great Recession, the Federal Reserve and, especially, Congress declared victory far too early. Congress even adopted “austerity” policies to reduce the federal debt when unemployment remained elevated and household finances fragile. Then later the Federal Reserve raised interest rates prematurely. The result was a long, painful recovery that took years longer than necessary and scarred many workers and the overall economic growth.

The $2 trillion American Rescue Package passed in March 2021 brought the total fiscal aid within a year to around $5 trillion or nearly a quarter of US GDP. It was the package that never came after the Great Recession. It was massive and did, in fact, help fuel a sharp pick-up in demand, along with vaccinations and re-openings. The Fed, too, continues to support a rapid, inclusive recovery. It is a bumpy road with an unexpectedly strong rebound in prices, as well as some employers finding it difficult to hire workers and producers coming up short on some supplies. Nonetheless, it is clear that we are on a faster path to recovery than in “jobless” recoveries after the prior two recessions.

The takeaway for policymakers and their advisers for the next crisis is to act forcefully at the start and stay the course until we have a recovery for all. It is worth it.

## Good policies require effective administration

Too often, relief during the coronavirus crisis did not reach all people who it was designed to help. Those groups who had the hardest time accessing the relief were often the ones who needed it most. Signing ceremonies for the legislation and victory laps by politicians due to the generous aid mean little to the people who got nothing or who struggled to get that aid. Administrative problems are not new, and they plague nearly every aid program in the United States, even outside of recessions. Policymakers must grapple with administrative failings and build systems that will serve all people in the next crisis.

Unemployment insurance suffered the most catastrophic failures during the COVID-19 crisis (Pancotti, 2021). Decades of neglect and in some cases deliberate attacks on the state-run programs made it vulnerable. In addition, Congress passed legislation to expand the measures and make weekly benefits more generous. Outdated technology systems made it impossible to raise replacement rates of workers' prior pay. Most states were only able to add a flat amount. Congress first chose $600, which would fully replace the typical unemployed workers pay. But that meant that about half of workers got more in benefits than they had earned before being laid off. That kicked off a debate about disincentives to return to work and labor shortages. It raged on and eventually led 26 Republican Governors to cut off the extra federal aid months before Congress had enacted it. Some of the largest states like Texas have unemployment rates well above the national average. Moreover, people of color are more likely to be unemployed.

Another example of a well-intended, new policy that suffered from administrative problems was the Payroll Protection Program. The goal of the program was to loan money to small business owners so they could continue to keep their employees on the payroll. If businesses did so, then their loans were converted to grants and they did not have to repay them. It is great policy, but it was run through the Small Business Administration, a small agency, and banks, who have a profit motive rather than a public policy mission. As a result of weak administrative systems, the program failed to live up to expectations.

Research by Doniger and Kay (2021) at the Federal Reserve shows that delays in the roll out of the Payroll Protection Programs reduced the number of employees whose jobs could have been saved. Specifically, they estimate that 1% fewer delayed loans would have lowered the unemployment rate by one-tenth percentage point. The delays and other administrative failings of the program affected the smallest businesses, the self-employed, lower paid, and Black- and Hispanic-owned businesses most. So again, the people who were least likely to get the relief were often the ones in most need of it. While millions of

Figure 3

**Six states ending jobless benefits early have unemployment above national average**

State unemployment rate in June 2021, seasonally adjusted

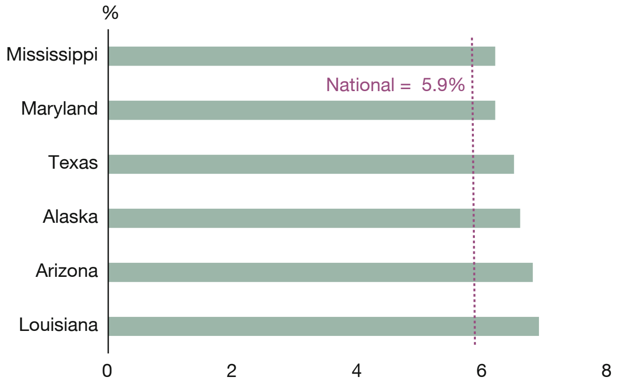

Source: Bureau of Labor Statistics.

businesses and employees benefited from the program, it must be improved to accomplish its goals equitably.

Solutions exist. The most effective one would be to put fiscal relief to families, the employed and small businesses on autopilot. Tying extra support to economic conditions – such that they start as soon as a recession begins and would continue until recovery is complete – would take some of the politics out of fighting recessions. For example, I argued for automatic stimulus checks to families in recessions and recoveries (Sahm, 2019). Automatic stabilizers for enhanced jobless benefits are in numerous legislative proposals and were endorsed by President Biden. This approach would allow policymakers to focus their energies on fighting the novel features of the crisis, whether a pandemic or underwater mortgage borrowers. Committing to deliver a basic set of relief programs would also force the government to build the administrative capacity for the programs. Congress enacts a set of policies in nearly every recession. We know what programs work. We know what programs are used. We can avoid the fire drill and administrative failings by making a commitment before the next recession.

More broadly, we learned that near universal cash relief is effective. Research shows that the three rounds of stimulus checks to most families provided both relief and boosted economic activity (Sahm, 2021a). In contrast, targeted programs are complicated and will often miss those in need of relief in recessions, because families' financial situations may deteriorate rapidly. The government has no systems to track which families are suffering hardship and which are not. Moreover, an accepted definition of what qualified as hardship does not exist, especially in recessions, which are widespread events. Cash relief, specifically for nearly all children in the so-called child tax credit, shows that policymakers see the benefits of the approach. The Biden Administration has proposed extending the one-year program for another five years. This is the first program – with no tax filing or work requirements – to serve families with children. It has the potential to cut the number of children living in poverty, especially deep poverty, but only if it is administered well.

The crisis magnified the existing shortcomings of our safety net. The failure of unemployment insurance went well beyond the administration of benefits. Experts have known for years that limited eligibility, geographic differences across states and insufficient amounts and duration of benefits were problematic, too. Numerous policy proposals exist, some of which have been laid out by West et al. (2016). What is lacking is the political will to reform the system and an acknowledgement across the political spectrum that the unemployed deserve aid, not derision, during recessions and expansions alike.

## Macroeconomists must upgrade their tools and frameworks

During the first year of the COVID-19 crisis, Congress enacted almost $5 trillion in fiscal relief and the Federal Reserve pumped trillions more into the financial system. Economists played a key role in shaping and debating this massive push to stabilize the economy. Many programs, like stimulus checks and extra weekly jobless benefits, were supersized. Several new programs like the payroll loans and Fed loans to municipalities were put together on the fly. Economists were instrumental – albeit far from unified – in crafting the big and bold policy response.

Economists, along with other policy experts, government staff and data scientists, have led efforts to evaluate the effects of the policies, often as soon as the first data become available. Studies like the one by Ganong et al. (2020) found that the extra $600 per week in jobless benefits did not create meaningful work disincentives. The Census Bureau created two new surveys of households and small businesses to track the rapidly changing economic conditions. Private data also offered timely insights on labor market conditions and consumer spending. The flood of studies and data was overwhelming at times, and as with any topic, research often differed in the findings. Even so, policymakers in Congress, the Administration, and the Federal Reserve had more information than in past crises to craft effective, evidence-based policies.

Arguments made by economists in the policy debates, especially surrounding the American Rescue Plan, also showed that their tools need an upgrade. A key example is the heavy reliance on aggregate statistics like GDP, the savings rate or personal income to evaluate the need for aid. Metrics such as potential output hide the wide differences across families. Decades of rising inequality in the United States have magnified the shortcomings of aggregate data. In fact, data from the Bureau of Economic Analysis, which disaggregates national personal disposable income shows that in 2018 (the latest available data) the top 10% of US households had as much personal income as the bottom 70%. The gap has risen since the Great Recession.

Figure 4

**Income inequality was large and rising before the pandemic**

Average household personal disposable income in 2007 and 2018, dollars, by income decile

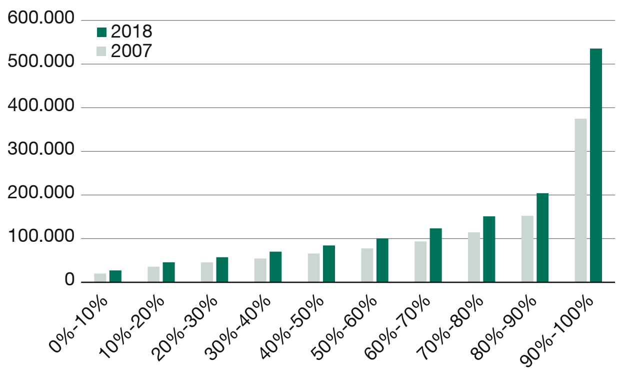

Source: Bureau of Economic Analysis.

Moreover, while the fiscal relief was generous by historical standards, it is trivial compared to the pre-crisis inequality. This helps explain why personal income – and aggregate – is higher now than before the pandemic, but the unemployment rate – in which each individual counts equally – remains nearly three percentage points higher and we have seven million fewer jobs than before. Aggregate statistics are useful in economic debates, but they need to be combined with other data that capture the heterogeneity in the US economy.

Another example of a longstanding framework that led some of the economic policy discussions astray is potential output. It is an estimate using methods developed in the 1960s of the economic capacity of the economy. How much demand could the businesses meet without inflation getting out of hand? While we can measure GDP, potential output to which GDP is compared is never observable. Nonetheless, economists like Blanchard (2021) used estimates from the Congressional Budget

Office to argue against the Rescue Plan. He treated the estimates of potential output as if they were a precise reality. Policy that hinges crucially on things we cannot see is problematic. Making matters worse, the official estimates assume that the racial inequalities in the labor market are the best we can do. Specifically, as I discuss in Sahm (2021b), the estimates of potential output assume a Black unemployment rate of 10%, the level achieved in 2005, as the lowest sustainable level of unemployment for Black workers.

More broadly, economists have wasted precious time during the coronavirus crisis using and defending falsified models. Empirics and common sense must sort out the old models that do not work and bring in new models that do. An upgrade, not a deletion of economic frameworks is necessary. Inexcusably, the Great Recession and its painfully slow recovery did not lead to an economics upgrade. As a result, the COVID-19 crisis showed once again how much intellectual baggage the economics profession carries with it. In large part, the falsified models lost out and new, bold policies were enacted. That is good, but it would be even better to have robust, evidence-based models to guide these new policies. In some cases, like the new, near-universal child benefits, policy is running ahead of our economic understanding. That program is essentially universal basic income for children, but the potential macroeconomic, long-term effects of such cash transfers have hardly been studied. Flogging the "dead parrots" of macro models incurs opportunity costs. It is time that would be better spent on creating new, lively models.

Better tools and frameworks do exist. The most important shift relative to the Great Recession is the economic views about the federal debt. Debt-to-GDP targets, such as those advanced by Reinhart and Rogoff (2011), served as an intellectual justification for austerity measures in the United States and Europe. Cutbacks in government spending slowed the recovery and led to lasting damage to workers and economic growth. During the COVID-19 crisis, centrist economists moved away from a limit to the level of debt and, as Furman and Summers (2020) argued, began to focus on debt service relative to GDP. The idea being that the country must make its debt payments but does not have to pay off the debt. Other economists further to the left, like Kelton (2020), a leader of the Modern Monetary Theory, argue that debt (in any form) should not set a limit on fiscal policy. Instead, the correct limit is inflation, and deficit spending could continue until the economy begins to overheat. Even five years ago, such a shift in the debate over the financing of fiscal policy was unthinkable, but it remains unclear which is the economic framework that will emerge and whether it will be central to policymaking.

Introspection among economists about the federal debt is one of many examples where collaboration across groups would lead to the best outcomes. Other areas that could benefit from an upgrade are how to design the social safety net, including unemployment insurance, so it supports people in need and overall economic growth. Models that incorporate differences across people, such as Heterogeneous Agent New Keynesian models, should be integrated into policy debates (Kaplan et al., 2018). Taking those models from academic journals to the halls of Congress will take considerable effort. They are complex, often black-box models, but they improve on aggregate models that are less and less effective given the inequality in the economy.

Finally, economists need to grapple more with the concept of full employment and the fact that the US economy has likely been operating below its potential for decades. Most economic models assume that – after a shock like a recession – the economy naturally moves back to a full employment equilibrium. However, other macroeconomic models, such as stock-flow consistent models (Nikiforos and Zezza, 2017), do not assume self-healing dynamics. The need for fiscal and monetary policy is notably different if the economy is perpetually operating below full employment. Again, it is not clear that one model is better than the other, but both bring useful insights. Unfortunately, debates about frameworks and tools often become contentious and unproductive. A true sea change in economic policy will require that economists embrace and promote change.

## References

[CR1] Bhutta, N., A. C. Chang, L. J. Dettling and J. W. Hsu (2020, 28 September), Disparities in Wealth by Race and Ethnicity in the 2019 Survey of Consumer Finances, *FEDS Notes*.

[CR2] Blanchard, O. (2021, 18 February), In defense of concerns over the $1.9 trillion relief plan, *PIIE Real Time Economic Issues Watch*.

[CR3] Cox, J. (2020, 16 April), Weekly jobless claims hit 5.245 million, raising monthly loss to 22 million due to coronavirus, *CNBC*.

[CR4] Doniger, C. and B. Kay (2021), Ten Days Late and Billions of Dollars Short: The Employment Effects of Delays in Paycheck Protection Program Financing, Finance and Economics Discussion Series, 2021-003, Board of Governors of the Federal Reserve System.

[CR5] Furman, J. and L. Summers (2020), *A Reconsideration of Fiscal Policy in the Era of Low Interest Rates*, Discussion Draft, Brookings.

[CR6] Ganong, P., P. Noel and J. S. Vavra (2020), US Unemployment Insurance Replacement Rates During the Pandemic, *BFI Working Paper*. 10.1016/j.jpubeco.2020.104273 PMC7525248 33012869

[CR7] Gould, E. and M. Kassa (2021), *Low-wage, low-hours workers were hit hardest in the COVID-19 recession*, Economic Policy Institute.

[CR8] Heggeness, M. L., J. Fields, Y. A. García Trejo and A. Schulzetenberg (2021), *Tracking Job Losses for Mothers of School-Age Children During a Health Crisis*, U.S. Census Bureau.

[CR9] Kaplan G, Moll B, Violante G (2018). Monetary Policy According to HANK. American Economic Review.

[CR10] Kelton, S. (2020), *The Deficit Myth: Modern Monetary Theory and the Birth of the People’s Economy*, PublicAffairs.

[CR11] Larrimore, J., J. Mortenson and D. Splinter (2021, 29 June), *Earnings Shocks and Stabilization During COVID-19*, 10.2139/ssrn.3876745 (8 July 2021). 10.1016/j.jpubeco.2021.104597 PMC8730490 35013626

[CR12] Mankiw, G. (2020, 15 April), *Two significant U.S. macroeconomic needs to consider amid the coronavirus pandemic*, Washington Center for Equitable Growth.

[CR13] Nikiforos, M. and G. Zezza (2017), Stock-flow Consistent Macroeconomic Models: A Survey, *Levy Economics Institute Working Paper*, 891.

[CR14] Pancotti, E. (2021), Unemployment Insurance in the pandemic, and beyond, The Weeds podcast, Vox.

[CR15] Parolin, Z., S. Collyer, M. A. Curran and C. Wimer (2021), *The Potential Poverty Reduction Effect of the American Rescue Plan*, Center on Poverty and Social Policy at Columbia University.

[CR16] Reinhart C M, Rogoff K (2011). From Financial Crash to Debt Crisis. American Economic Review.

[CR17] Sahm, C. (2019), *Direct Stimulus Payments to Individuals*, The Hamilton Project, Policy Proposal.

[CR18] Sahm (2020, 29 April), The coronavirus recession is severe, and the damage to the U.S. economy will last years, *Washington Center for Equitable Growth*.

[CR19] Sahm, C. (2021a), *They Worked: The effects of $1,400 stimulus checks on families and the economy*, Jain Family Institute.

[CR20] Sahm (2021b, 9 April), Fears of a Too Hot Economy Ignore Racial Inequality, *Bloomberg Opinion*.

[CR21] Spriggs, W. E. (2021), The Urgency of Now to Speed the Recovery, Testimony prepared for US House of Representatives Committee on Financial Services 117th Congress, First Session Hearing on “More than a Shot in the Arm: The Need for Additional COVID-19 Stimulus”.

[CR22] West, R., I. Dutta-Gupta, K. Grant, M. Boteach, C. McKenna and J. Conti (2016), Strengthening Unemployment Protections in America, Center for American Progress.

